# Switching of supramolecular nanostructures at the solid–liquid interface: interplay of bias polarity and solution concentration†

**DOI:** 10.1039/d5na00289c

**Published:** 2025-06-17

**Authors:** Baoxin Jia, Mihaela Enache, Bettina D. Gliemann, Angelina Jocic, Milan Kivala, Meike Stöhr

**Affiliations:** a Zernike Institute for Advanced Materials, University of Groningen Netherlands m.a.stohr@rug.nl; b Chair of Organic Chemistry I, Department of Chemistry and Pharmacy, Universität Erlangen-Nürnberg Germany; c Institute of Organic Chemistry, University of Heidelberg Germany milan.kivala@oci.uni-heidelberg.de; d University of Applied Sciences of the Grisons Switzerland

## Abstract

The self-assembly of a carboxy-functionalized triarylamine derivative (CTA) at the nonanoic acid–highly oriented pyrolytic graphite (NA–HOPG) interface is investigated using scanning tunnelling microscopy (STM). The study reveals that CTA molecules can self-assemble into diverse two-dimensional long-range ordered networks at the NA–HOPG interface, with their formation influenced by the concentration of the solution and the bias voltage of the STM tip. Moreover, reversible switching between the porous structures and the close-packed structure is induced by changing the bias polarity. We identify that for this phenomenon to occur the negatively polarised O atoms of the carboxylic groups of CTA play an important role, enabling the CTA molecules at the interface to desorb and re-adsorb which is essential for switching from one ordered arrangement to the other. Our findings demonstrate that reversible switching can be controlled by manipulating the solution concentration as well as the applied bias voltage, which holds promise for controlling switchable molecular systems at the solid–liquid interface.

## Abstract 2

Reversible switching between porous and close-packed supramolecular networks at the solid–liquid interface is controlled by both the applied bias polarity and solution concentration, offering a route to smart surfaces.

## Graphical abstract

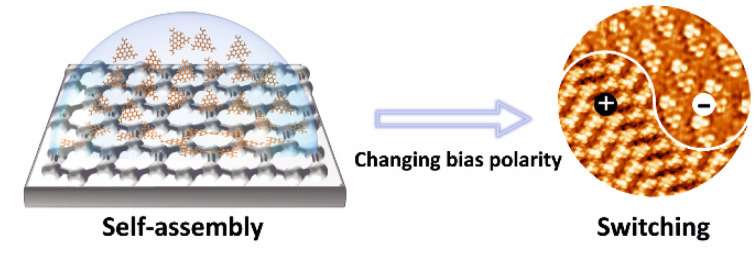

## Introduction

Supramolecular nanostructures have gained increasing interest in recent years, because of their possible applications in, for instance, life sciences, two-dimensional (2D) crystal engineering, and smart materials.^1–8^ Self-assembly provides an effective approach for fabricating supramolecular nanostructures in general^9–12^ and at solid–liquid interfaces in particular.^13–19^ Recent research has shown that 2D supramolecular self-assemblies at solid–liquid interfaces can exhibit structural transformations in response to external stimuli,^3,19–21^ a phenomenon known as “switching”. Stimuli that can induce switching include temperature,^22–24^ light,^25–31^ pH,^32,33^ ionic triggers,^34–36^ and electric fields.^37–50^ This controlled switching between different molecular structures at the solid–liquid interface presents a promising strategy for developing smart surfaces, which holds potential applications in the fields of molecular switches, molecular motors, biological sensors, and more.^3,15,19–21^

Carboxylic groups attached to molecular building blocks enable the formation of supramolecular nanostructures at the solid–liquid interface *via* hydrogen bonding.^52–54^ Some of these supramolecular nanostructures exhibit switching behaviour induced by an external electric field at the solid–liquid interface.^39–48,50,51^ Two well-known examples are the aromatic carboxylic acids, benzene-1,3,5-tricarboxylic acid (trimesic acid, TMA)^39,43–46^ and 1,3,5-tris(4-carboxyphenyl)-benzene (BTB).^39–41,47^ Previous studies have demonstrated that both TMA and BTB can form various hydrogen-bonded molecular self-assemblies at the highly oriented pyrolytic graphite (HOPG)–liquid interface, depending on the applied bias voltage. In addition, for both molecules, reversible phase transformations between different molecular self-assemblies could be induced by changing the polarity of the bias voltage. Mostly, the switching behaviour has been investigated using solutions of one fixed concentration. However, a few reports mentioned that solution concentration can influence the switching behaviour. Cometto *et al.*^40^ observed, for BTB at the nonanoic acid (NA)–HOPG interface, differences in the switching behaviour upon varying the solution concentration: the phase transformation from the porous to the compacted phase occurred more slowly in highly diluted solutions than in saturated ones. Nonetheless, the influence of concentration on the switching mechanism was not further explored. In another study, Deng *et al.*^47^ reported, for BTB at the octanoic acid–HOPG interface, bias-induced switching between the chicken-wire, oblique, and compact phases. In particular, they found that the switching pathway depended on the solution concentration: in low-concentration solutions, BTB transformed from the compact phase at positive bias to the chicken-wire structure and subsequently to the oblique phase upon applying a negative bias voltage. In contrast, in high-concentration solutions, the transformation occurred directly from the compact to the oblique phase upon changing the bias polarity. Additionally, the influence of temperature and the presence of water at the interface on the switching process has also been investigated.^45,46^ Although the switching of carboxy-functionalized molecules induced by external electric fields has been studied for years, the mechanism of this phenomenon is still under debate. Different assumptions, including deprotonation of the carboxylic groups, formation of dipole moments, and the presence of negatively polarised O atoms in the carboxylic groups, have been suggested to explain this phenomenon in previous studies.^40,41,43–46^ Therefore, continuous exploration of aromatic carboxylic acids capable of demonstrating switching behaviour at the solid–liquid interface and in-depth investigations into the diverse factors influencing this phenomenon, such as the polarity and absolute value of the applied bias voltage, solution concentration, and the structural attributes of the switching molecule, remain prominent study questions in the field of surface science.

Scanning tunnelling microscopy (STM) is a powerful technique for investigating the structure of molecular assemblies at the solid–liquid interface. It provides direct insight into the supramolecular structures with (sub)molecular resolution and enables the control and manipulation of molecular assemblies *via* the STM tip.^11–15,55,56^ Since the applied bias polarity can be changed in a highly controlled manner using STM, STM is an ideal tool for investigating reversible switching between different molecular nanostructures at interfaces.^37–51^

**Fig. 1:**
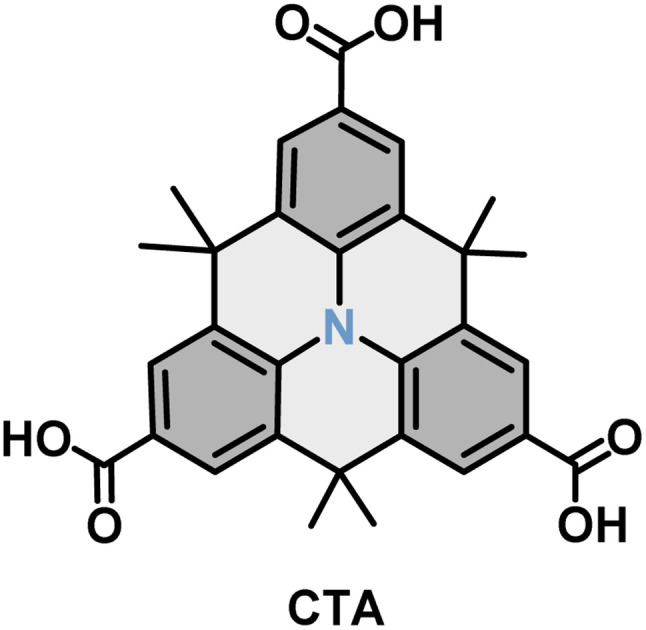
Schematic molecular structure of 4,4,8,8,12,12-hexamethyl-4*H*,8*H*,12*H*-benzo[1,9]quinolizino[3,4,5,6,7-*defg*]acridine-2,6,10-tricarboxylic acid (CTA).

In this work, the self-assembly of a carboxy-functionalized triarylamine derivative, 4,4,8,8,12,12-hexamethyl-4*H*,8*H*,12*H*-benzo[1,9]quinolizino[3,4,5,6,7-*defg*]acridine-2,6,10-tricarboxylic acid (CTA, see Fig. 1), at the NA–HOPG interface was explored by STM. CTA has 3-fold symmetry and consists of a planar triarylamine core to which 3 carboxylic groups are attached at each corner. On each side, the triarylamine core is bridged by dimethylmethylene tethers, which are oriented out of the plane formed by the triarylamine core. The carboxylic groups can form directional hydrogen-bonds, which may permit the formation of self-assembled networks. Steiner *et al.* reported that CTA molecules assembled into porous hydrogen-bonded networks on Au(111) under ultrahigh vacuum (UHV) conditions.^57^ It should be noted that under UHV conditions, switching of the assemblies upon changing the bias voltage has so far not been reported. However, the self-assembly of CTA at the solid–liquid interface, as well as possible switching behaviour induced by changing the polarity of the applied bias voltage, has not yet been studied.

We show that CTA molecules formed both porous and close-packed hydrogen-bonded networks at the NA–HOPG interface. The formation of the respective type of network was found to be influenced by the solution concentration as well as the applied bias polarity. Moreover, we found reversible switching between the porous and close-packed networks induced by changing the tip-sample bias polarity *in situ*. The switching process was found to be affected by the solution concentration and the magnitude of the applied bias voltage, with the speed of switching also determined by these factors. Based on these results, we identified that the switching mechanism involves the negatively polarised O atoms of the carboxylic groups of CTA, which leads to the adsorption or desorption of these molecules at the interface depending on the bias polarity. As a result, the number of CTA molecules at the interface increases or decreases, resulting in the formation of different bias-dependent structures. Our findings demonstrate that the switching process can be controlled by manipulating the solution concentration and applied bias voltage, which holds promise for engineering further switching systems at the solid–liquid interface based on our findings.

## Experimental

CTA was synthesized by oxidation of the previously reported three-fold formylated triarylamine (compound S1, see Scheme S1†)^58^ using chromium(vi) oxide and concentrated sulfuric acid (for the detailed synthetic procedure and characterization, see the ESI†). CTA molecules were dissolved in nonanoic acid (Sigma-Aldrich, 96%, CAS number 112-05-0) and solutions with three different concentrations were prepared: saturated, 50% saturated and 20% saturated. For this, first, a slightly oversaturated solution of CTA in nonanoic acid was prepared. The saturated solution was obtained by drawing liquid from the top of the oversaturated solution kept in a vial. The molar concentration of the slightly oversaturated CTA solution (≈573.1 μM) was taken as the reference value for the saturated solution. A 50% saturated solution (≈286.6 μM) was prepared by mixing 1 ml saturated solution and 1 ml nonanoic acid. A 20% saturated solution (≈114.6 μM) was prepared by mixing 1 ml saturated solution and 4 ml nonanoic acid. All the solutions were sonicated (Branson 1510) for 5 min. A HOPG (SPI Supplies, SPI-2 ZYB) crystal was used as the substrate. The HOPG crystal was cleaved using adhesive tape before every measurement. For each STM measurement, a droplet of the CTA solution was placed on the HOPG substrate. All STM experiments were performed at the solid–liquid interface under ambient conditions using a Molecular Imaging Keysight N9700C scanner operated in constant-current mode. STM tips were prepared by mechanical cutting from a Pt/Ir (90 : 10) wire (Goodfellow, 0.25 mm diameter). All voltages are given with respect to a grounded tip. All STM images were analysed and processed using WSxM 5.0.^59^ The unit cell values of the different CTA phases were derived by averaging over approximately 30 high-resolution STM images.

## Results

### Molecular phases of CTA molecules at the NA–HOPG interface

**Fig. 2:**
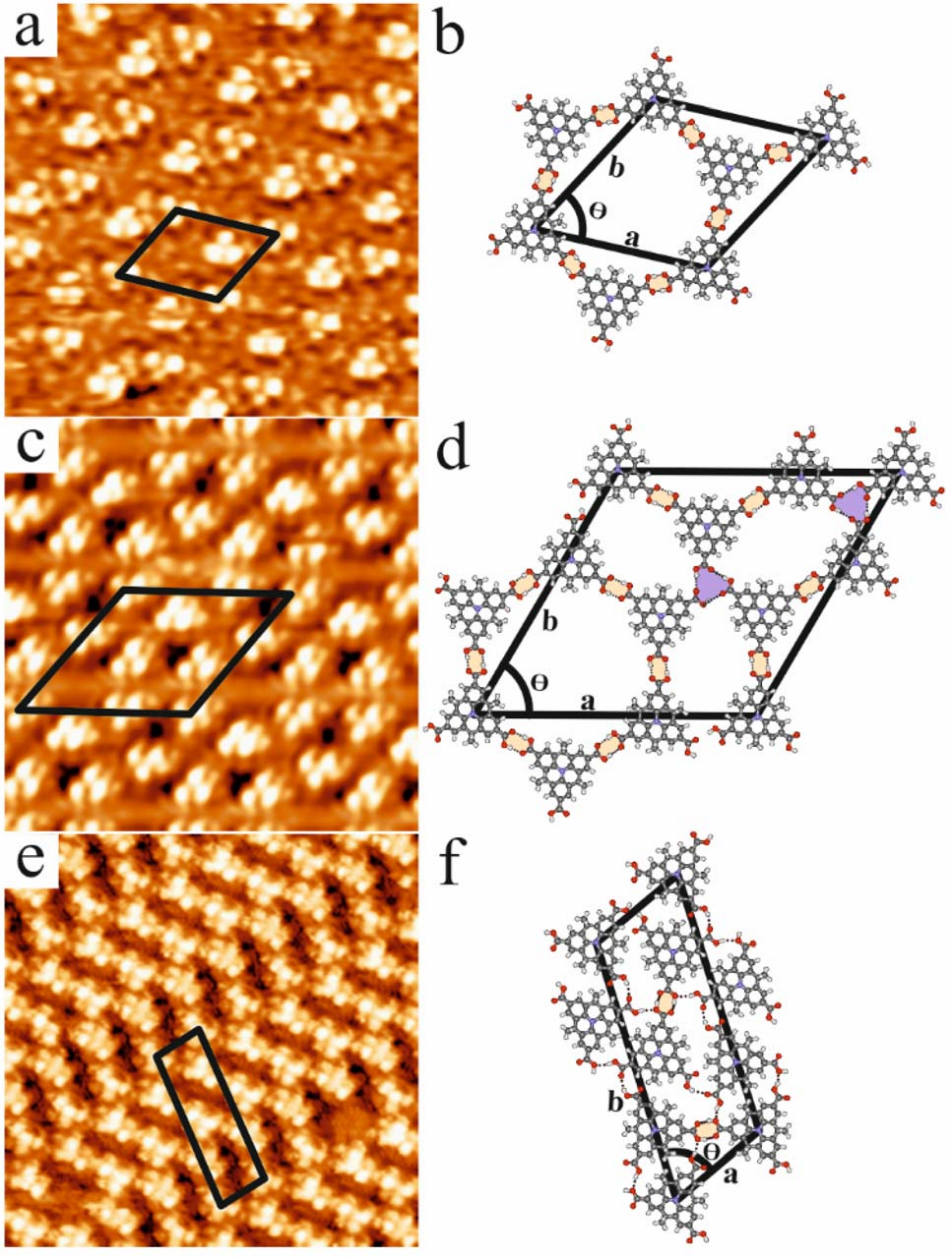
(a) STM image (10 × 10 nm^2^, *V*_bias_ = −1.0 V, *I* = 20 pA, saturated solution) showing the CTA chicken-wire structure at the NA–HOPG interface. (b) Molecular model of the chicken-wire structure which is stabilized by dimeric O–H⋯O hydrogen bonds (highlighted in yellow and shown by black dotted lines). The unit cell is indicated by a black rhombus. (c) STM image (10 × 10 nm^2^, *V*_bias_ = −1.0 V, *I* = 20 pA, 50% saturated solution) showing the CTA flower structure at the NA–HOPG interface. (d) Molecular model of the flower structure which is stabilized by dimeric O–H⋯O (highlighted in yellow) hydrogen bonds as well as cyclic trimeric (highlighted in blue) hydrogen bonds. The intermolecular O–H⋯O hydrogen bonds are indicated by black dotted lines. The unit cell is indicated by a black rhombus. (e) STM image (10 × 10 nm^2^, *V*_bias_ = +1.0 V, *I* = 20 pA, fully saturated solution) showing the CTA close-packed structure at the NA–HOPG interface. (f) Molecular model of the close-packed structure which is stabilized by single (indicated by dotted lines) hydrogen bonds as well as dimeric O–H⋯O hydrogen bonds (highlighted in yellow and shown by black dotted lines). The unit cell is indicated by a black parallelogram.

The self-assembly of CTA at the NA–HOPG interface was investigated by STM. It was found that CTA molecules can self-assemble into three different well-ordered networks depending on both the solution concentration and the bias voltage. Fig. 2 summarizes the STM images of these three structures and also shows their tentative molecular models. Fig. 2a shows the first structure (chicken-wire structure) that CTA molecules can self-assemble into, which was obtained by using a saturated solution of CTA in NA with a sample bias of −1.0 V. The intramolecular features of CTA can be distinguished in this high resolution STM image. Each CTA molecule appears as three bright protrusions in the STM image corresponding to the out-of-plane configuration of the methyl groups with respect to the triarylamine core. The carboxy groups, which typically yield comparatively weak contrast and are more difficult to identify in STM images,^57,59,60^ are located, directionally speaking, between the methyl groups. Thus, the rotational adsorption configuration of a single molecule can be easily identified based on the location of the three methyl groups.^61^Fig. 2b shows the molecular model of the chicken-wire structure that is stabilized by dimeric O–H⋯O H-bonds between the terminal carboxy groups (highlighted in yellow). Thereby, each CTA molecule interacts with three neighbouring molecules *via* dimeric H-bonds and a porous chicken-wire structure is formed, similar to the chicken-wire structure formed by other carboxy-functionalised molecules like BTB and TMA at the solution–HOPG interface.^39–50,62–68^ The unit cell parameters for the chicken-wire structure were determined from the STM data to be *a* = *b* = 2.6 ± 0.4 nm, and *θ* = 60 ± 6°. The molecular density for the chicken-wire structure is 0.34 molecules per nm^2^.

This chicken-wire structure was also observed for CTA molecules adsorbed on Au(111) at the dry interface under UHV conditions, and the unit cell parameters are similar.^57^ Besides the chicken-wire structure, similar to what has been reported for TMA molecules^43–45,64–68^ adsorbed at the solution–HOPG interface, CTA molecules can also form another porous structure (flower structure) at the NA–HOPG interface, which is shown in Fig. 2c. The STM image was obtained by using a 50% saturated solution of CTA in NA at a sample bias of −1.0 V. The flower structure consists of hexagonal units which also form the basis of the chicken-wire structure and which are made up of six molecules. Within a hexagonal unit, the CTA molecules interact *via* dimeric H-bonds between their carboxy groups. However, unlike in the chicken-wire structure, the flower structure includes trimeric H-bonds, each of which is formed between the carboxylic groups of three different molecules belonging to three neighbouring hexagonal units. Fig. 2d depicts the molecular model of the flower structure, which is stabilized by dimeric hydrogen bonds (highlighted in yellow) and cyclic trimeric hydrogen bonds (highlighted in blue). The unit cell parameters for the flower structure determined from the STM data are *a* = *b* = 4.2 ± 0.2 nm, and *θ* = 60 ± 5°. The molecular density of the flower structure is 0.39 molecules per nm^2^, which is slightly higher compared to the chicken-wire structure. Fig. 2e shows the third type of network (close-packed structure) that CTA can form at the NA–HOPG interface. Fig. 2e was obtained by using a saturated solution of CTA in NA at a sample bias of +1.0 V. Fig. 2f shows the molecular model of the close-packed structure. In this structure, CTA molecules interact with each other through a combination of single hydrogen bonds (indicated by single dotted lines) and dimeric O–H⋯O hydrogen bonds (highlighted in yellow) between the carboxylic groups. The unit cell was determined from the STM data with lengths *a* = 1.4 ± 0.2 nm and *b* = 4.0 ± 0.4 nm, and an internal angle of *θ* = 71 ± 4°. Compared to the chicken-wire and flower structures, the molecular density of the close-packed structure is much higher (0.76 molecules per nm^2^). Overview STM images of the three different CTA networks, including differently oriented domains, are shown in Fig. S1.†

Our findings as summarized in Fig. 2 suggest that the formation of the three different molecular networks is influenced by the solution concentration and bias voltage. Both of these effects on the formation of the molecular networks were investigated in detail and the outcomes of these investigations are detailed below.

### Influence of both the solution concentration and bias voltage on the different CTA networks

**Fig. 3:**
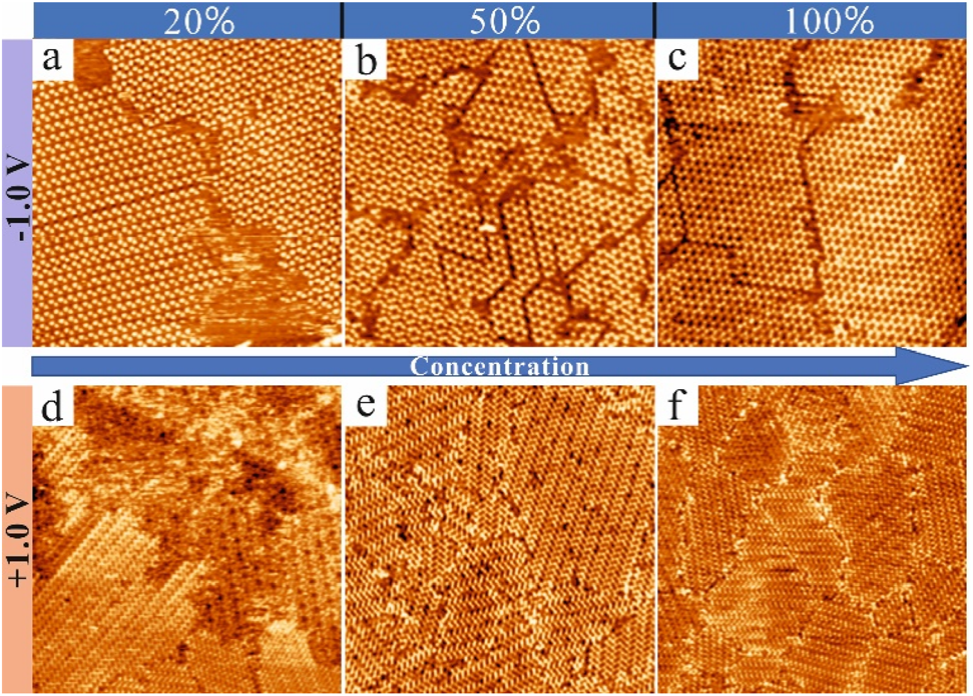
STM images (80 × 80 nm^2^, 20 pA) showing the concentration-dependent self-assembled arrangements of CTA at the interface between NA and HOPG. The STM images shown in (a–c) were taken with negative bias voltages while the ones displayed in (d–f) were taken with positive bias. At negative bias voltages and independent of the concentration, only the chicken-wire and flower structures were observed. On the other hand, at positive bias voltages, mainly the close-packed arrangement was observed and for the 20% saturated solution (c) the chicken-wire and flower structures were occasionally observed while the close-packed structure remained the dominant phase. The scanning conditions are (a) −1.0 V, 20% saturated solution; (b) −1.0 V, 50% saturated solution; (c) −1.0 V, saturated solution; (d) +1.0 V, 20% saturated solution; (e) +1.0 V, 50% saturated solution; (f) +1.0 V, saturated solution.

To investigate the effects of both the concentration of CTA in NA and the bias voltage on the network formation, three solutions of CTA in NA with different concentration were prepared: 20% saturated solution, 50% saturated solution, and saturated solution. The influence of solution concentration was investigated under both bias polarities. Fig. 3 summarizes the results of these studies. At negative bias voltages and independent of the concentration, only the porous structures (chicken-wire and flower structures) were observed. When the bias voltage was kept at −1.0 V, for the 20% and 50% saturated solutions, the chicken-wire and flower structure coexisted at the interface as shown in Fig. 3a and b, respectively. However, for the saturated solution, we only observed the chicken-wire structure as shown in Fig. 3c. On the other hand, when the bias voltage was kept at +1.0 V, for the 20% saturated solution, we observed that the chicken-wire, flower and close-packed structures coexisted (Fig. 3d). For the 50% saturated and the saturated solution, we only observed the close-packed phase as shown in Fig. 3e and f, respectively. Fig. 3 clearly shows that the concentration of CTA in NA has an obvious influence on the molecular networks formed by CTA at the NA–HOPG interface. Notably, from Fig. 3 it is already obvious that not only the solution concentration but also the bias polarity has an influence on the type of structure formed.

### Bias-induced switching between different CTA phases

**Fig. 4:**
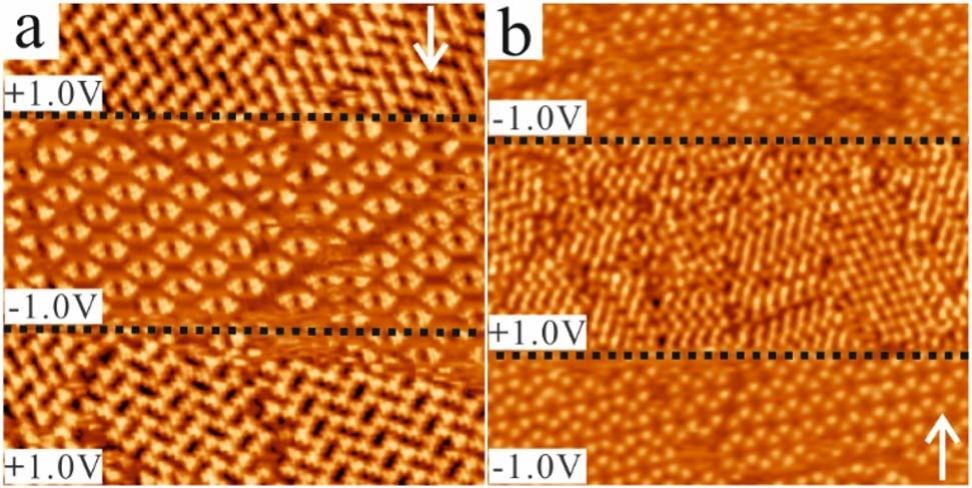
Bias-induced switching between different phases of CTA at the NA–HOPG interface for (a) the saturated and (b) the 50% saturated solution. The white arrows indicate the scan direction and the black dashed lines indicate the scan line at which the bias voltage was changed. (a) In this STM image (30 × 30 nm^2^, 20 pA), the successful reversible phase transformation from the close-packed structure into the chicken-wire structure and back can be seen. The transformation from one phase into the others happened almost instantaneously. (b) In this STM image (50 × 50 nm^2^, 20 pA), the phase transformation from the porous structure to the close-packed structure is demonstrated. The phase transformation also occurred for the less concentrated solution almost instantaneously.

**Fig. 5:**
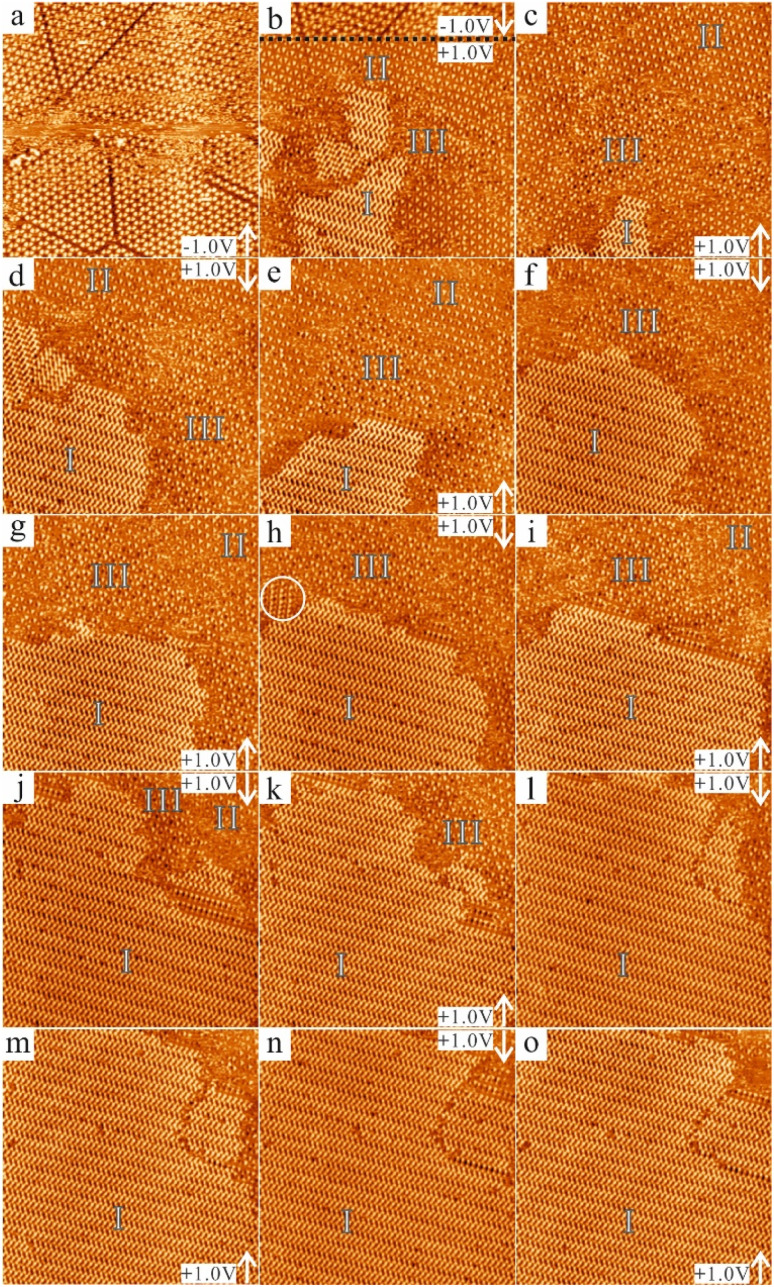
Sequential STM images (70 × 70 nm^2^, 20 pA) of the same area showing the delay of the phase transformation from the porous structures into the close-packed structure for the 20% saturated solution. The white arrows indicate the scan direction. The acquisition time of each image was 63 s. (a) CTA molecules formed the chicken-wire structure. The sample bias was kept at −1.0 V. (b) Upon changing the sample bias from −1.0 V to +1.0 V at the black dashed line, the chicken-wire structure did not exclusively exist anymore. Instead, co-existence of three structures can be seen: the close-packed structure (labelled as I), the chicken-wire structure (labelled as II) and the flower structure (labelled as III). (c–o) The sample bias was kept at +1.0 V. The white circle highlights a precursor structure for the close-packed structure. The STM images show that the molecules gradually rearranged into the close-packed structure and in (o), the close-packed structure almost exclusively occupies the whole scan region.

In a next step, the effect of changing the polarity of the bias voltage on the molecular self-assemblies of CTA was investigated. Additionally, we also investigated whether the concentration of CTA influenced this process. To study this, three solutions of CTA in NA were used for the STM measurements: saturated solution, 50% saturated solution, and 20% saturated solution. For each of them, the polarity of the bias voltage was changed reversibly during the performed STM measurements. The results are summarized in Fig. 4 and 5.

Fig. 4a presents a phase transformation upon changing the voltage polarity for the saturated solution. In Fig. 4a, along the scanning direction indicated by the white arrow, it can be clearly seen that when the bias voltage was changed from +1.0 V to −1.0 V and then back to +1.0 V (polarity changes are indicated by the black dashed line), the molecular arrangement of CTA changed from the close-packed to the chicken-wire structure and back to the close-packed structure. The changes occurred instantaneously. This means that a bias-induced phase transformation of CTA can be achieved at the NA–HOPG interface by changing the polarity of the bias voltage during the STM measurements, which is similar to the switching reported previously for carboxy-functionalized molecules like TMA,^39,42–46,48^ BTB^39–41,47^ and a 5-(benzyloxy)isophthalic acid derivative.^50^ For the 50% saturated solution, the switching behaviour was also observed. Fig. 4b shows that, along the scanning direction indicated by the white arrow, the two porous structures (chicken-wire and flower structures) observed at the beginning were transformed into the close-packed structure and back to one porous structure (chicken-wire structure) when the bias voltage was changed from −1.0 V to +1.0 V and back to −1.0 V (polarity changes are indicated by the black dashed line). Again, the changes occurred instantaneously. Thus, reversible bias-induced switching among the different structures of CTA can also be achieved at the NA–HOPG interface for the diluted solution. Besides, we observed that the flower structure, which co-existed at the beginning with the chicken-wire structure, was transformed into the close-packed structure after switching the bias from −1.0 V to +1.0 V, but reverted to the chicken-wire structure when the bias was switched back to −1.0 V. This indicates that the chicken-wire structure is preferred over the flower structure at negative sample bias.

Up to now, the switching was successfully accomplished by changing the polarity of the bias voltage for the saturated and 50% saturated solution. It is noted that the switching from the close-packed into the porous structures occurred for both concentrations instantaneously while the reverse instantaneous switching was only observed for the fully saturated solution. For the 50% saturated solution, both situations were observed: instantaneous switching as shown in Fig. 4b as well as delayed switching when the bias polarity was changed from negative to positive (Fig. S2†). This indicates that the solution concentration can influence the switching of CTA; there is a possibility of a delay in the phase transformation from the porous structures to the close-packed one in the low concentration solutions. The delayed switching behaviour was consistently observed across different samples and STM tips. This reproducibility suggests that the phenomenon is neither tip-related nor resolution dependent.

The delay was always observed for the 20% saturated solution. As shown in Fig. 5, switching also occurred when changing the bias polarity, but a delay in the phase transformation from the porous structures to the close-packed one was observed, while no delay was detected in the reverse direction. As can be seen in the STM image displayed in Fig. 5a, the CTA molecules self-assembled into the chicken-wire structure at a sample bias of −1.0 V. In the consecutive STM image (Fig. 5b), the same scanning conditions were maintained for the topmost part and the chicken-wire structure was still present. At the black dashed line, the sample bias was then switched to +1.0 V. However, in contrast to the fully saturated and 50% saturated solution, as shown in Fig. 4, the phase transformation into the close-packed structure did not take place immediately. Instead, only after some time, the first small domains of close-packed networks (labelled I) appeared and they co-existed with the chicken-wire and flower structures (labelled II and III, respectively). As shown in the consecutive STM images in Fig. 5c–o, the domain sizes of both the chicken-wire and flower networks were reduced and finally disappeared with time, whereas the domain sizes of the close-packed networks increased and finally became dominant across the whole scan area. It should be noted that the transformation did not happen gradually. That is, the domain sizes of the two porous structures did not gradually decrease while that of the close-packed structure gradually increased. Instead, both increases and decreases in the areas of the three different structures between consecutive images were observed. During the transformation, a precursor structure of the close-packed structure was observed, which was found to be metastable and finally transformed into the close-packed structure (indicated by the white circle in Fig. 5h). Besides, rotationally different domains of the close-packed structure were present at the beginning of the phase transformation (Fig. 5b and d) which merged into one large domain with time. This observation indicates that the formation of large ordered close-packed networks is driven by the Ostwald ripening process during the transformation.^68–73^ One interesting fact is that this delayed switching only occurs when the bias polarity is changed from negative to positive. For both the saturated and diluted solution, the switching from the close-packed to the porous structure is always instantaneous whereas the inverse switching direction shows a delay for the diluted solution. The influence of the solution concentration provides indications for understanding the mechanism underlying the switching, which will be discussed in the next session.

**Fig. 6:**
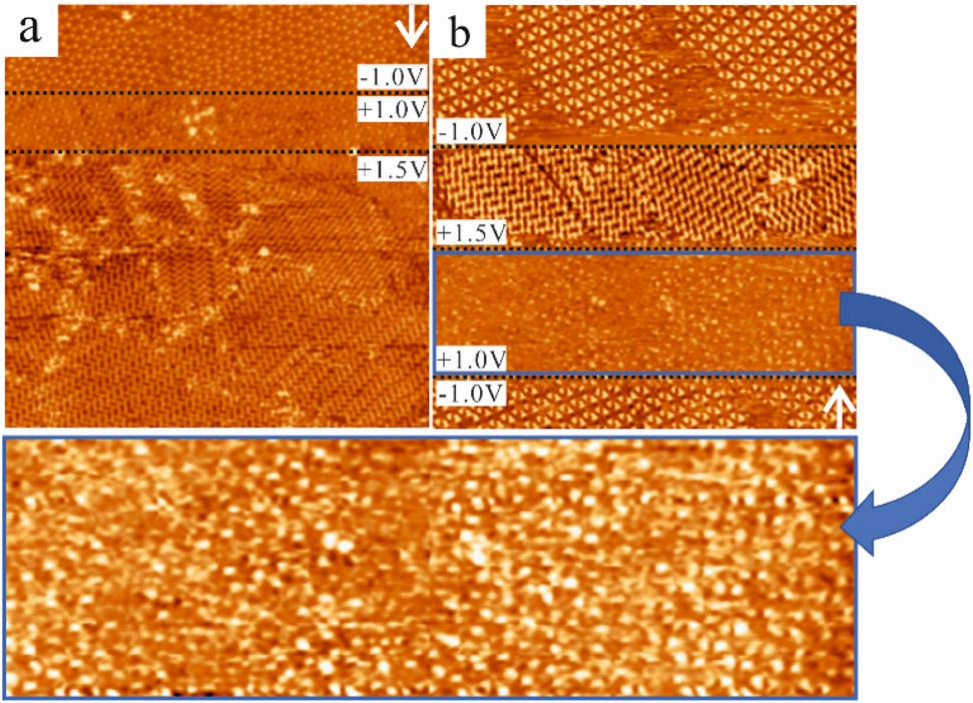
The magnitude of the bias voltage influences the switching of CTA at the NA–HOPG interface for (a) the 50% saturated and (b) the 20% saturated solution. The white arrows indicate the scan direction and the black dashed lines indicate at which scan line the bias voltage was changed. (a) In this STM image (80 × 80 nm^2^, 20 pA), the porous structure did not transform into the close-packed structure instantaneously after switching the bias to +1.0 V. When the sample bias was increased to +1.5 V, the CTA molecules rearranged into the close-packed structure instantaneously. (b) The STM image (80 × 80 nm^2^, 20 pA) shows a similar process as in (a), where the molecules did not instantaneously rearrange into the close-packed structure upon changing the bias voltage from −1.0 V to +1.0 V. However, the rearrangement happened instantaneously when the bias voltage was increased to +1.5 V. The region in the blue rectangle represents an enlarged view of the area in (b) scanned at +1.0 V. The presence of the porous structure can be identified.

It was found that the magnitude of the bias voltage also influenced the switching behaviour. A higher positive bias voltage (compared to the value required to observe the switching behaviour for the saturated solution) can promote the transformation from the porous into the close-packed structure in the diluted solution. Fig. 6 shows that for both the 50% saturated and the 20% saturated solution, the phase transformation from the porous structures into the close-packed structure did not happen instantaneously after switching the bias from −1.0 V to +1.0 V, but upon changing the bias voltage value to +1.5 V, the CTA molecules rearranged into the close-packed structure instantaneously. Therefore, the absolute value of the bias voltage is also important to induce the phase transformation of CTA at the NA–HOPG interface and increasing the absolute value can accelerate the transformation from the porous structures into the close-packed structure.

In addition to the solution concentration and the magnitude of the bias voltage, the ambient humidity also has an influence on the switching behaviour. As STM measurements at the solid–liquid interface are conducted under ambient conditions, the presence of trace amounts of water or other impurities in the air can potentially impact the results. So far, this influence has not been explicitly addressed in previous studies. We explored the influence of ambient humidity on the switching behaviour of CTA through a series of STM measurements. The results in Fig. 4–6 were obtained when the humidity was around 50% on average. Under these conditions, the delayed phase transformation of CTA from the porous structures to the close-packed structure was observed only in the diluted solution. However, when the humidity decreased to around 20% on average, this delayed phase transformation also occurred in the saturated solution. Thus, ambient humidity can influence the switching behaviour of CTA at the NA–HOPG interface.

To explore the role of water in the system, the saturated CTA solution was mixed with Milli-Q water in a 1 : 1 ratio. It was observed that the saturated CTA solution and water formed two distinct liquid phases: one constituted the CTA solution, while the other appeared as a milky phase where some CTA molecules dissolved into water. This observation suggests that an excess of water might not be favourable for the molecular self-assembly of CTA at the NA–HOPG interface. In further investigations, a droplet of Milli-Q water was added to the saturated CTA solution to assess its impact on the switching behaviour. No significant effects were observed, and the switching from the chicken-wire structure to the close-packed structure remained delayed. Inspired by the work of Saeed *et al.*^45^ who reported that water can facilitate the switching of TMA at the octanoic acid–water film/HOPG interface we performed a similar experiment. Saeed *et al.* created a water film on HOPG by exposing the HOPG sample to water vapour before adding the solution of TMA molecules in octanoic acid. Bias-induced switching between the chicken-wire and close-packed phases of TMA was observed in their case. In our experiments, we covered the HOPG sample with a water film using the same method. However, no significant influence on the switching of CTA was observed. The switching from the chicken-wire to the close-packed structure remained delayed.

In a last method, we tried to directly increase the humidity to ∼60% using a humidifier. In this case, the STM measurements revealed instantaneous switching from the chicken-wire to the close-packed structure. Conversely, decreasing the humidity back to ∼20% resulted again in delayed switching. These observations clearly indicate that humidity, specifically the concentration of water in the air, effectively influences the switching behaviour of CTA. Another noteworthy result is that the delayed switching at low humidity can become instantaneous when a larger positive bias voltage is applied. This observation indicates an interplay between humidity and the applied bias voltage in adjusting the CTA switching behaviour at the NA–HOPG interface.

In summary, the STM bias voltage can induce switching between the different CTA structures at the NA–HOPG interface, whereas solution concentration, the magnitude of the bias voltage and environmental humidity are additional parameters influencing the switching. The forthcoming section will delve into the mechanism underlying these intriguing phenomena.

## Discussion

### The influence of concentration on the formation of CTA phases

Since our results indicate that the solution concentration of CTA in NA influences the formation of CTA self-assembled structures, which is also bias-dependent, the influence of concentration at the different voltage polarities is discussed separately.

At positive bias voltage, the close-packed structure formed for the high-concentration solutions (saturated and 50% saturated solution), while the porous structures and close-packed structure coexisted in the low concentration regime (20% saturated solution). Similar observations have been reported for other carboxy-functionalized molecules at the solution–HOPG interface,^40,47,66^ indicating a preference for more densely packed phases for higher concentrations. We tentatively assign our observations to the negatively polarised O atoms of the carboxy groups of CTA. Consequently, the adsorption of CTA molecules near the interface is influenced by the polarity of the applied bias. When a positive bias voltage is applied to the sample, CTA molecules are attracted to the NA–HOPG interface because of the negatively polarised O atoms of their carboxy groups. The more concentrated the solution is, the more molecules can be adsorbed at the interface. In the high-concentration solutions, there are enough molecules available to form the close-packed structures and cover the whole HOPG surface and thus, the close-packed structure is exclusively observed for these solutions. In contrast, (very) low-concentration solutions do not provide enough molecules comparatively to completely cover the surface with the close-packed structure. As a result, less dense structures (porous structures) are formed in certain regions. Hence, for low-concentration solutions, a mixture of close-packed and porous structures is observed. Consequently, at positive bias voltages, the close-packed structure is exclusively observed for high-concentration solutions, whereas for low-concentration solutions a mixture of close-packed and porous structures is usually observed.

At negative bias voltage and for saturated solutions, only one type of porous structure was observed, namely the chicken-wire structure. Rarely, a surprising coexistence of the two porous structures (chicken-wire and flower structures) was noted at the interface for the diluted solutions. Since the flower structure has an around 15% higher molecular density compared to the chicken-wire one, it should not be present according to our above reasoning. However, one should keep in mind that the basic building block of the chicken-wire and flower structures is the same, a pore consisting of 6 CTA molecules and only the interlinking of these porous units differs. Thus, from a statistical point of view the probability of the formation of the flower structure is not zero. Additionally, the presence of the solvent could also influence and/or stabilize the observed patches of the flower structure. The CTA molecules form porous structures, which are generally stabilized by the co-adsorption of solvent molecules.^40,43^ Ochs *et al.* reported that the co-adsorption of solvent molecules influences the formation of porous phases, while the size and shape of the pores are determined by the co-adsorbed solvent molecules.^75^ Lastly, we would like to point out that the CTA flower structure was observed for the diluted solution only for a short period of approximately three months. That suggests that its formation could be kinetically driven and/or that it is a metastable phase. Compared to the chicken-wire structure, it has a higher molecular density and thus, it could be, based on this argument, slightly favoured from an energetic point of view. However, it features a combination of double and single intermolecular H-bonds compared to only double intermolecular H-bonds present in the chicken-wire structure. From this point of view, the chicken-wire structure is preferred. Based on these considerations, it cannot be easily deduced which structure should be energetically favoured. Unfortunately, the exact parameters for the occurrence of the flower structure could not be determined.

### The mechanism underlying the switching behaviour

The switching of CTA can be induced by changing the bias polarity, which is further influenced by the solution concentration, bias magnitude and environmental humidity. By changing the bias polarity, a reversible phase transformation between the CTA porous structures and the close-packed structure could be induced. While the phase transformation from the close-packed structure to the porous structures always occurred instantaneously, the phase transformation from the porous structures to the close-packed structure was delayed in diluted solutions and at low humidity. The delayed phase transformation could be accelerated by adjusting the voltage magnitude. Up to now, similar bias-induced switching has been reported for several carboxy-functionalized molecules at the solid–liquid interface and the following possible explanations have been proposed: (1) partial deprotonation of the terminal carboxy groups,^41,45,46^ (2) formation of a dipole moment^40,76^ and (3) interaction of the negatively polarised O atoms of the carboxy groups with the biased sample surface.^43^ We will discuss each of these mechanisms in turn.

The first assumption presumes a partial deprotonation of the carboxy groups in solution to elucidate the bias-induced switching between different assembly structures of carboxy-functionalized molecules. According to this assumption, the deprotonated carboxy-functionalized molecules rearrange upon changing the polarity of the bias voltage, resulting in a phase transformation between a densely packed and a porous structure.^41^ While this explanation has shown promise in elucidating the switching behaviour of TMA molecules with the aid of water, polar additives, or thermal treatment,^45,46,77^ its universality has not yet been shown. Notably, in the case of a 5-(benzyloxy)isophthalic acid derivative at the 1-octanol/HOPG interface, switching was observed even when an additive inhibiting the deprotonation of carboxy groups was present in the solution.^50^ This finding weakens the explanation relying solely on the partial deprotonation of the carboxy groups. For our work, the deprotonation assumption fails to account for the influence of solution concentration on the switching behaviour. The delayed phase transformation of CTA in diluted solutions contradicts the expectation of instantaneous switching if partial deprotonation were to occur. Additionally, the observation of the Ostwald ripening process during the phase transformation of CTA molecules cannot be explained by this assumption. Consequently, this assumption offers a limited explanation for understanding the bias-induced switching at the solid–liquid interface.

The second explanation, first proposed by Cometto *et al.*,^40^ is based on the alignment of a molecular dipole with respect to the applied external electric field when reversing the direction of the applied bias to explain the bias-induced switching of BTB at the NA–HOPG interface.^40^ However, this mechanism also cannot fully explain the results observed in this study. Firstly, the CTA molecules form planar hydrogen-bonded networks at the NA–HOPG interface, making it improbable to possess a molecular dipole. Secondly, the possible existence of the dipole moment does not account for the observed influence of solution concentration, bias magnitude, and humidity on the switching of CTA, particularly the delayed switching phenomenon of CTA. These inconsistencies between the predictions of the dipole alignment model and our experimental findings raise doubts about its applicability to this system. Therefore, despite being considered as an explanation in previous research, the alignment of a molecular dipole with respect to the electric field does not provide a comprehensive understanding of the bias-induced switching of CTA molecules at the NA–HOPG interface.

According to the third explanation, the bias-induced switching can be attributed to the presence of negatively polarised O atoms of the carboxy groups and their interaction with the biased sample surface. This point of view was elucidated by Ubink *et al.* in their study on the switching behaviour of TMA molecules at the NA–HOPG interface.^43^ Since TMA possesses negatively polarised O atoms at the carboxy groups, its behaviour at the interface is influenced by the polarity of the sample bias. Under a positive sample bias, TMA molecules are attracted to the interface, maximizing the number of adsorbed molecules, resulting in the formation of dense structures. On the other hand, under a negative sample bias, some TMA molecules desorb into the solution, while others assemble into porous structures at the interface. These porous structures are stabilized by the co-adsorption of solvent molecules within the pores and their interactions with the substrate. Consequently, a phase transformation was observed when the polarity of the bias voltage was changed.

Similarly, the bias-induced switching between CTA structures is consistent with the third explanation. For the positive sample bias, the CTA molecules are attracted to the interface, maximizing the number of adsorbed molecules. Thus, the close-packed structure with higher molecular density is formed. When the bias voltage polarity is changed to negative, some CTA molecules desorb into the solution, while others remain at the interface and rearrange into porous structures. These porous structures are stabilized at the interface through the co-adsorption of solvent molecules and interactions with the substrate. If the bias voltage polarity is reverted to positive, additional CTA molecules from the solution will adsorb at the interface once more, prompting all molecules at the interface to rearrange into the close-packed structure and covering the entire interface. Consequently, the observed phase transformation occurs as a result of changes in voltage polarity. This mechanism hinges on the adsorption and desorption processes, as well as the rearrangement of CTA molecules at the interface, which are all influenced by the polarity of the bias voltage. Furthermore, this elucidates the role of the negatively polarised O atoms of the carboxy groups in driving the bias-induced switching between the different CTA structures at the solid–liquid interface.

Based on the above-described mechanism, the influence of solution concentration, voltage magnitude, and environmental humidity on the switching process can be explained. Notably, the delayed phase transformation only occurs in the direction from porous to close-packed structures and never in the reverse direction. As previously discussed, the adsorption and desorption of CTA molecules at the interface depend on the polarity of the bias voltage. When the transformation from porous to close-packed structures takes place, additional CTA molecules need time to adsorb at the interface. The rate of this adsorption process (molecules per s per nm^2^) governs the overall time required for the transformation. This adsorption rate can be affected by the solution concentration, voltage magnitude, and environmental humidity, thereby leading to the delayed phase transformation. The effects of each factor are discussed in turn below.

The solution concentration generally determines the adsorption rate of molecules at the interface.^74,78^ Higher concentrations result in faster adsorption rates, thus influencing, in the present case, the speed of the phase transformation. The delayed phase transformation observed for the diluted solutions is associated with the adsorption and desorption of CTA molecules. During the phase transformation from the close-packed to the porous structures, some CTA molecules desorb, which, however, cannot be observed by STM. The remaining molecules at the interface are sufficient to form the porous structures and their rearrangement is instantaneous. During the transformation from the porous structures to the close-packed one, the CTA molecules already present at the interface begin to rearrange into the close-packed structure. Simultaneously, further CTA molecules from the solution adsorb at the interface. This process directly depends on the solution concentration and the lower concentration causes the lower adsorption rates. Gradually, the newly adsorbed CTA molecules also arrange into the close-packed networks. During this process, some adsorbed CTA molecules may initially form porous structures but ultimately rearrange into close-packed structures through Ostwald ripening. This can cause fluctuations in the domain size of the three CTA structures as presented in Fig. 5. Eventually, the close-packed networks enlarge and become the exclusive structure at the interface. The lower adsorption rate of CTA molecules in diluted solutions prolongs the adsorption process, while in saturated solutions, the higher adsorption rate results in faster switching. Therefore, the delay in the phase transformation is observed in diluted solutions but not in saturated ones.

The influence of environmental humidity on the adsorption rate of CTA molecules is not as straightforward as the effects of solution concentration and bias magnitude. Firstly, the results indicate that water molecules in air have a more significant impact on the switching process than when added to the CTA solution or when using a sample covered with a water film. These results indicate that water molecules in air more easily influence the switching process than in liquid. The water molecules in air surrounding the droplet applied to the HOPG sample most likely have a lower diffusion barrier into the CTA solution (towards the interface) compared to the water molecules in the liquid state. The intermolecular interactions among water molecules in the liquid state, primarily hydrogen bonds, make them more tightly bound. Thus, it can be possibly inferred that water molecules influence the switching process when they dissolve into the CTA solution and form a water–solution system. Secondly, it should be noted that CTA monomers are more likely to contribute to the self-assembly process at the interface compared to CTA molecules aggregated in solution. Previous studies^67,79^ have shown that pre-aggregated solute molecules can exist in the solution, which do not take part in the self-assembly process at the interface. In this work, pre-aggregated CTA molecules may also be present in the solution. When a trace amount of water dissolves in the solvent, it aids in “fully dissolving” these pre-aggregated CTA molecules. Upon exposure to ambient conditions, a homogeneous water–solution system forms, which is more polar than the pure CTA solution. As CTA molecules contain polar carboxy groups, they preferentially dissolve in a more polar environment. Consequently, the water–solution system promotes the dissolution of pre-aggregated molecules, increasing the concentration of CTA monomers in the solution. Compared to low humidity, high humidity favours the diffusion of water molecules from the air into the CTA solution. More water molecules can diffuse into the CTA solution at higher humidity, which results in a higher CTA monomer concentration. The increased monomer concentration enhances the adsorption rate of CTA molecules, resulting in a faster phase transformation from porous to close-packed structures. Consequently, the switching process becomes instantaneous at high humidity. In contrast, at low humidity, the effects of the water–solution system are less pronounced, leading to a lower concentration of CTA monomers in the solution. This lower monomer concentration leads to a reduced adsorption rate of CTA molecules, resulting in a delayed switching process from porous to close-packed structures.

The voltage magnitude can also influence the adsorption rate of CTA. The CTA molecules are attracted to the interface at positive sample bias due to the negatively polarised O atoms of their carboxy groups. As the positive bias voltage increases, the adsorption rate of CTA molecules increases proportionally due to the increased electric field. Therefore, by applying a larger positive voltage value, the adsorption process of CTA can be effectively promoted. Consequently, in diluted solutions or under low humidity conditions, where the adsorption rate might otherwise be lower, increasing the magnitude of the positive voltage can facilitate the rapid adsorption of CTA molecules at the interface, leading to an instantaneous transformation from porous to close-packed structures.

## Conclusions

STM measurements revealed that the CTA molecules formed three distinct structures at the NA–HOPG interface. Two are porous structures (chicken-wire and flower structures) and one is a close-packed structure. The formation of these structures was influenced by both sample bias and solution concentration.

The solution concentration influenced, depending on the bias polarity, the formation of CTA structures. At negative bias, porous arrangements were observed. However, since the flower structure was found to form occasionally, the solution concentration cannot be ascribed as the reason for the formation of either the chicken-wire or the flower structure. At positive bias and relatively high concentrations, close-packed structures were present. In very diluted solutions, both porous and close-packed structures coexisted, as the number of CTA molecules was insufficient to exclusively cover the interface with the close-packed structure.

The reversible switching between the porous CTA structures and the close-packed structure could be accomplished by changing the polarity of the applied bias voltage. This was in turn influenced by the solution concentration, voltage magnitude and environmental humidity. The observed bias-induced switching behaviour is largely attributed to the (un)favourable interaction of the negatively polarised O atoms of the carboxy groups with the biased sample surface. Moreover, the solution concentration, voltage magnitude and environmental humidity influence the adsorption rate of the CTA molecules at the interface and can result in a delayed phase transformation from the porous structures to the close-packed structure.

This work demonstrates that the bias-induced switching of carboxy-functionalized molecules at the solid–liquid interface is closely related to the presence of polarised functional groups. The switching can be controlled not only by manipulating the solution concentration and applied bias voltage but is also influenced by the ambient environment, highlighting its potential for designing switchable surfaces at the solid–liquid interface or even under ambient conditions.

## Author contributions

M. E. and M. S. conceived and directed the experiments. B. J. conducted the STM measurements and performed the data analysis. M. E. provided support for the experiments and data analysis. B. D. G. and A. J. synthesised and characterised the compounds. M. K. provided support for the synthesis. B. J. wrote the original draft. All authors contributed to the discussion of the results and the writing of the manuscript.

## Conflicts of interest

There are no conflicts to declare.

## Supplementary Material

NA-007-D5NA00289C-s001

## Data Availability

The authors confirm that all data supporting the results and conclusions reported in this study are available within the paper and the ESI.† Additional data used for the study are available from the corresponding authors upon reasonable request.

## References

[cit1] Aida T., Meijer E. W., Stupp S. I. (2012). Science.

[cit2] Elemans J. A. A. W., Lei S., De Feyter S. (2009). Angew. Chem., Int. Ed..

[cit3] Zhang X., Zeng Q., Wang C. (2013). Nanoscale.

[cit4] Mali K. S., Pearce N., De Feyter S., Champness N. R. (2017). Chem. Soc. Rev..

[cit5] Sosa-Vargas L., Kim E., Attias A. J. (2017). Mater. Horiz..

[cit6] Shen X., Song J., Sevencan C., Leong D. T., Ariga K. (2022). Sci. Technol. Adv. Mater..

[cit7] Lim J. Y. C., Lin Q., Xue K., Loh X. J. (2019). Mater. Today Adv..

[cit8] Gontero D., Lessard-Viger M., Brouard D., Bracamonte A. G., Boudreau D., Veglia A. V. (2017). Microchem. J..

[cit9] Li M., Schnablegger H., Mann S. (1999). Nature.

[cit10] Whitesides G. M., Grzybowski B. (2002). Science.

[cit11] Lehn J. M. (2002). Science.

[cit12] Philp D., Stoddart J. F. (1996). Angew. Chem., Int. Ed..

[cit13] De Feyter S., De Schryver F. C. (2003). Chem. Soc. Rev..

[cit14] De Feyter S., De Schryver F. C. (2005). J. Phys. Chem. B.

[cit15] Ferreira Q., Delfino C. L., Morgado J., Alcácer L. (2019). Materials.

[cit16] Teyssandier J., De Feyter S., Mali K. S. (2016). Chem. Commun..

[cit17] Furukawa S., De Feyter S. (2008). Top. Curr. Chem..

[cit18] Ciesielski A., Palma C. A., Bonini M., Samorì P. (2010). Adv. Mater..

[cit19] Sahare S., Ghoderao P., Chana Y., Lee S. L. (2023). Nanoscale.

[cit20] Peng X., Zhao F., Peng Y., Li J., Zeng Q. (2020). Soft Matter.

[cit21] Elemans J. A. A. W. (2016). Adv. Funct. Mater..

[cit22] Blunt M. O., Adisoejoso J., Tahara K., Katayama K., Van der Auweraer M., Tobe Y., De Feyter S. (2013). J. Am. Chem. Soc..

[cit23] Gutzler R., Sirtl T., Dienstmaier J. F., Mahata K., Heckl W. M., Schmittel M., Lackinger M. (2010). J. Am. Chem. Soc..

[cit24] Cheng L., Li Y., Zhang C. Y., Gong Z. L., Fang Q., Zhong Y. W., Tu B., Zeng Q., Wang C. (2016). ACS Appl. Mater. Interfaces.

[cit25] Tao Shen Y., Deng K., Zhang X. M., Feng W., Zeng Q., Wang C., Gong J. R. (2011). Nano Lett..

[cit26] Yokoyama S., Hirosea T., Matsuda K. (2014). Chem. Commun..

[cit27] Wieghold S., Li J., Simon P., Krause M., Avlasevich Y., Li C., Garrido J. A., Heiz U., Samorì P., Müllen K., Esch F., Barth J. V., Palma C. A. (2016). Nat. Commun..

[cit28] Garah M. El., Borré E., Ciesielski A., Dianat A., Gutierrez R., Cuniberti G., Bellemin-Laponnaz S., Mauro M., Samorì P. (2017). Small.

[cit29] Frath D., Yokoyama S., Hirose T., Matsuda K. (2018). J. Photochem. Photobiol., C.

[cit30] González J. D. C., Iyoda M., Rabe J. P. (2018). Angew. Chem., Int. Ed..

[cit31] Bonacchi S., El Garah M., Ciesielski A., Herder M., Conti S., Cecchini M., Hecht S., Samorì P. (2015). Angew. Chem., Int. Ed..

[cit32] Piot L., Meudtner R., El Malah T., Hecht S., Samorì P. (2009). Chem.–Eur. J..

[cit33] Li H., Xu X., Shang J., Li J., Hu X., Teo B. K., Wu K. (2012). J. Phys. Chem. C.

[cit34] Cui K., Mali K. S., Wu D., Feng X., Müllen K., Walter M., De Feyter S., Mertens S. F. L. (2017). Small.

[cit35] Hirsch B. E., McDonald K. P., Qiao B., Flood A. H., Tait S. L. (2014). ACS Nano.

[cit36] Cucinotta A., Kahlfuss C., Minoia A., Eyley S., Zwaenepoel K., Velpula G., Thielemans W., Lazzaroni R., Bulach V., Hosseini M. W., Mali K. S., De Feyter S. (2023). J. Am. Chem. Soc..

[cit37] Lei S., Deng K., Yang Y., Zeng Q., Wang C., Jiang J. (2008). Nano Lett..

[cit38] Mali K. S., Wu D., Feng X., Müllen K., Van der Auweraer M., De Feyter S. (2011). J. Am. Chem. Soc..

[cit39] Velpula G., Teyssandier J., De Feyter S., Mali K. S. (2017). ACS Nano.

[cit40] Cometto F. P., Kern K., Lingenfelder M. (2015). ACS Nano.

[cit41] Lee S.-L., Fang Y., Velpula G., Cometto F. P., Lingenfelder M., Müllen K., Mali K. S., De Feyter S. (2015). ACS Nano.

[cit42] Zheng Q.-N., Liu X.-H., Liu X.-R., Chen T., Yan H.-J., Zhong Y.-W., Wang D., Wan L.-J. (2014). Angew. Chem., Int. Ed..

[cit43] Ubink J., Enache M., Stöhr M. (2018). J. Chem. Phys..

[cit44] Cometto F. P., Arisnabarreta N., Vanta R., Jacquelin D. K., Vyas V., Lotsch B. V., Paredes-Olivera P. A., Patrito E. M., Lingenfelder M. (2024). ACS Nano.

[cit45] Saeed M., Mahmood A., Saleemi A. S., Zeng X., Lee S.-L. (2020). J. Phys. Chem. C.

[cit46] Mahmood A., Saeed M., Chan Y., Saleemi A. S., Guo J., Lee S.-L. (2019). Langmuir.

[cit47] Deng C., Liu Z., Ma C., Zhang H., Chi L. (2020). Langmuir.

[cit48] Li W., Xu S., Chen X., Xu C. (2021). Chin. Chem. Lett..

[cit49] Cai Z.-F., Zhan G., Daukiya L., Eyley S., Thielemans W., Severin K., De Feyter S. (2019). J. Am. Chem. Soc..

[cit50] Li S.-Y., Yang X.-Q., Chen T., Wang D., Wang S.-F., Wan L.-J. (2019). ACS Nano.

[cit51] Hong Y., Wang L., Wang S.-F., Wang D., Chen T. (2021). CrystEngComm.

[cit52] Kudernac T., Lei S., Elemansa J. A. A. W., De Feyter S. (2009). Chem. Soc. Rev..

[cit53] Ivasenko O., Perepichka D. F. (2011). Chem. Soc. Rev..

[cit54] Lackinger M., Heckl W. M. (2009). Langmuir.

[cit55] Münninghoff J. A. W., Elemans J. A. A. W. (2017). Chem. Commun..

[cit56] Zhang X., Chen Q., Deng G.-J., Fan Q.-H., Wan L.-J. (2009). J. Phys. Chem. C.

[cit57] Steiner C., Gliemann B. D., Meinhardt U., Gurrath M., Meyer B., Kivala M., Maier S. (2015). J. Phys. Chem. C.

[cit58] Gliemann B. D., Strauss V., Hitzenberger J., Dral P. O., Hampel F., Gisselbrecht J.-P., Drewello T., Thiel W., Guldi D. M., Kivala M. (2017). Chem.–Eur. J..

[cit59] Horcas I., Fernández R., Gómez-Rodríguez J. M., Colchero J., Gómez-Herrero J., Baro A. M. (2007). Rev. Sci. Instrum..

[cit60] Gottardi S., Müller K., Moreno-López J. C., Yildirim H., Meinhardt U., Kivala M., Kara A., Stöhr M. (2014). Adv. Mater. Interfaces.

[cit61] Müller K., Moreno-López J. C., Gottardi S., Meinhardt U., Yildirim H., Kara A., Kivala M., Stöhr M. (2016). Chem.–Eur. J..

[cit62] Silly F. (2012). J. Phys. Chem. C.

[cit63] Kampschulte L., Lackinger M., Maier A.-K., Kishore R. S. K., Griessl S., Schmittel M., Heckl W. M. (2006). J. Phys. Chem. B.

[cit64] Griessl S., Lackinger M., Edelwirth M., Hietschold M., Heckl W. M. (2002). Single Mol..

[cit65] Lackinger M., Griessl S., Heckl W. M., Hietschold M., Flynn G. W. (2005). Langmuir.

[cit66] Shi H., Lu X., Liu Y., Song J., Deng K., Zeng Q., Wang C. (2018). ACS Nano.

[cit67] Ha N. T. N., Gopakumar T. G., Hietschold M. (2011). J. Phys. Chem. C.

[cit68] Ha N. T. N., Gopakumar T. G., Hietschold M. (2013). Surf. Sci..

[cit69] Gong J.-R., Lei S.-B., Wan L.-J., Deng G.-J., Fan Q.-H., Bai C.-L. (2003). Chem. Mater..

[cit70] Stabel A., Heinz R., De Schryver F. C., Rabe J. P. (1995). J. Phys. Chem..

[cit71] Ahn S., Matzger A. J. (2012). J. Am. Chem. Soc..

[cit72] Piskorz T. K. (2018). et al.. J. Phys. Chem. C.

[cit73] Piskorz T. K., de Vries A. H., De Feyter S., van Esch J. H. (2019). J. Phys. Chem. C.

[cit74] Gurdumov K., Mazur U., Hipps K. W. (2022). J. Phys. Chem. C.

[cit75] Ochs O., Hocke M., Spitzer S., Heckl W. M., Martsinovich N., Lackinger M. (2020). Chem. Mater..

[cit76] Cometto F., Frank K., Stel B., Arisnabarreta N., Kernac K., Lingenfelder M. (2017). Chem. Commun..

[cit77] Mahmood A., Zeng X., Saleemi A. S., Cheng K.-Y., Lee S.-L. (2020). Chem. Commun..

[cit78] Gurdumov K., Mazur U., Hipps K. W. (2022). J. Phys. Chem. C.

[cit79] Velpula G., Martin C., Daelemans B., Hennrich G., Van der Auweraer M., Mali K. S., De Feyter S. (2021). Chem. Sci..

