# Supplementary material for: Switching of supramolecular nanostructures at the solid–liquid interface: interplay of bias polarity and solution concentration
Source: Nanoscale Adv. 2025 Jun 17;7(16):4897–907. doi: 10.1039/d5na00289c (PMC12208213; doi:10.1039/d5na00289c)
Supplement: NA-007-D5NA00289C-s001 [file NA-007-D5NA00289C-s001.pdf]

Supplementary Information (SI) for Nanoscale Advances.
This journal is © The Royal Society of Chemistry 2025

Supporting Information for

Switching of supramolecular nanostructures at the solid-liquid interface: interplay of bias polarity and solution concentration

Baoxin Jia,[a] Mihaela Enache,[a] Bettina D. Gliemann,[b] Angelina Jocic,[c] Milan Kivala,*[c] Meike Stöhr,*[a,d]

a. Zernike Institute for Advanced Materials, University of Groningen, Netherlands. Email: m.a.stohr@rug.nl
b. Institute of Organic Chemistry, University of Heidelberg, Germany.
c. Chair of Organic Chemistry I, Department of Chemistry and Pharmacy, Universität Erlangen-Nürnberg, Germany
d. University of Applied Sciences of the Grisons, Switzerland.

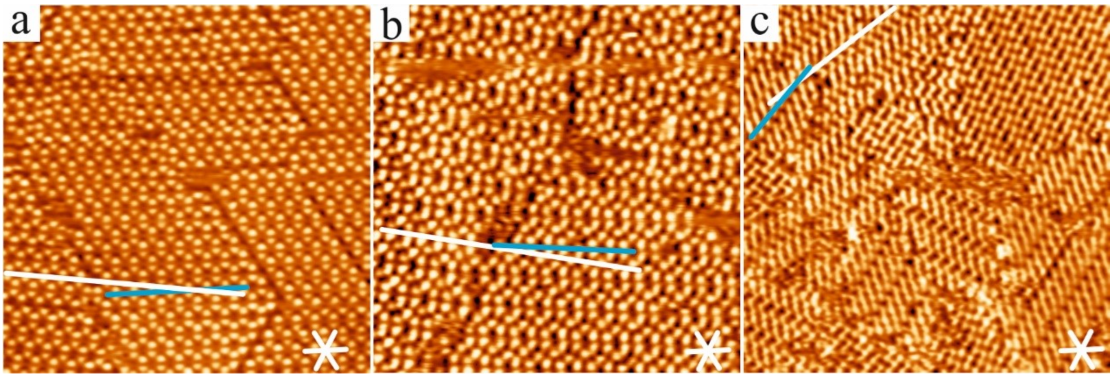

Figure S1 (a) STM image (50 x 50 nm^2, V_{bias} =-1.0 V, I = 20 pA, 50% saturated solution) showing the chicken-wire structure formed by CTA molecules at the interface between NA and HOPG. (b) STM image (50 x 50 nm^2, V_{bias} =-1.0 V, I = 20 pA, 50% saturated solution) showing the flower structure formed by CTA molecules at the interface between NA and HOPG. (c) STM image (50 x 50 nm^2, V_{bias} =1.0 V, I = 20 pA, 50% saturated solution) showing the close-packed structure formed by CTA molecules at the interface between NA and HOPG. The white and blue solid lines highlight two differently oriented rotational domains. The hexagonal white star indicates the orientation of the HOPG substrate.

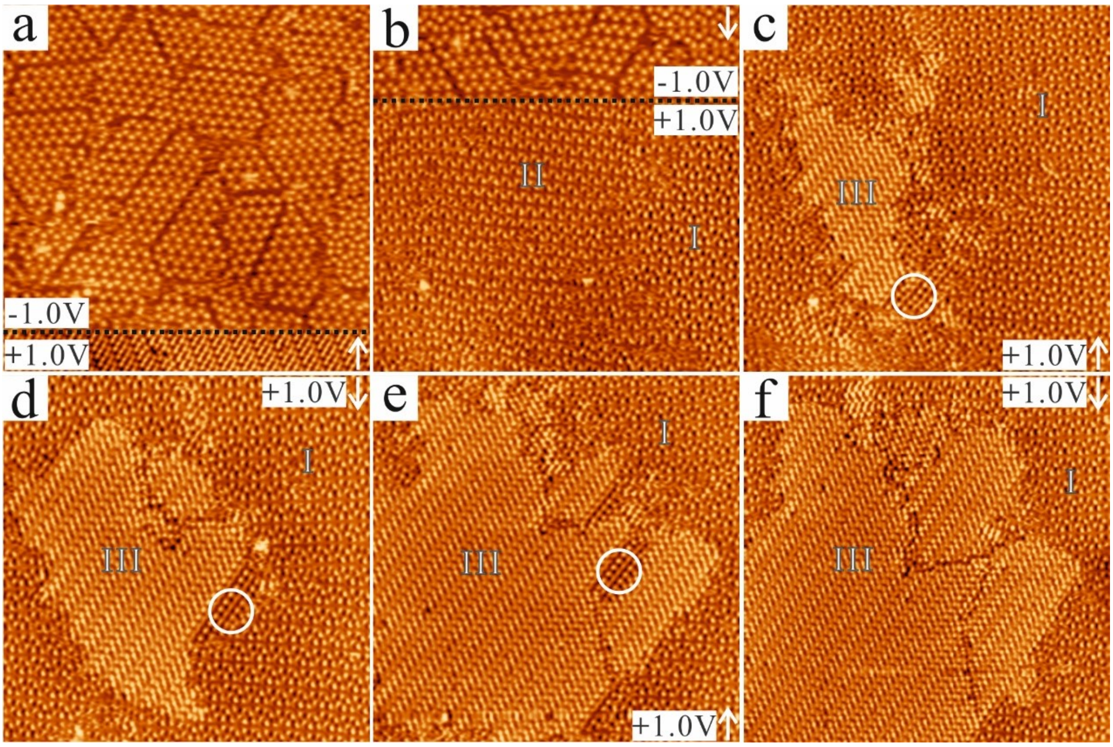

Figure S2 Sequential STM images (70 x 70 nm^2, 20 pA) of the same area showing the delay of the phase transformation from the porous structures into the close-packed structure for a 50% saturated solution. The white arrows indicate the scan direction. The acquisition time of each image was 84s. (a) The sample bias was changed to -1.0 V at the black dashed line, after which the CTA molecules rearranged into the porous structures instantaneously. (b) Upon changing the sample bias from -1.0 V to +1.0 V (black dashed line), two porous structures (labelled as I and II respectively) did not transform into the close-packed structure instantaneously. (c-f) The sample bias was kept at +1.0 V. The molecules gradually rearranged into the close-packed structure (labelled as III). The close-packed structure coexisted with the porous structure and occupied more and more space gradually. The white circle highlights a precursor state for the close-packed structure.

Supplementary Methods

Materials and general methods

Solvents and reagents were purchased at reagent grade from commercial suppliers like Merck/Sigma Aldrich, TCI, Thermo Fisher Scientific, Acros Organics, and Honeywell and were used without further purification. Compound S1 was synthesized according to a literature protocol published by Kivala and coworkers.[1] Analytical TLC was performed on aluminum plates coated with 0.20 mm silica gel and a fluorescent indicator (Macherey-Nagel, ALUGRAM®, SIL G/U30V254). Components were visualized by exposure to ultraviolet light (λ = 254 nm and 366 nm). ^{1}H NMR and ^{13}C NMR spectra were recorded in $(CD_3)_2CO$ on a Bruker Avance 300 (Bruker, 300 MHz for ^{1}H) and Bruker Avance 400 (Bruker, 101 MHz for ^{13}C). Chemical shifts (δ) are reported in parts per million (ppm) and were referenced to the residual solvent signal as an internal reference (($(CD_3)_2CO$: 2.05 ppm for ^{1}H; CD_3OD: 49.0 ppm for ^{13}C).[2] Coupling constants (*J*) are given in Hz and the apparent resonance multiplicity is reported as s (singlet), d (doublet), t (triplet), or m (multiplet). Mass spectrometry (MS) analysis was measured on a 6220 oaTOF (Agilent Technologies, ESI) in the mass spectrometry department of the Organic Chemical Institute, Friedrich-Alexander University of Erlangen-Nürnberg. Infrared (IR) spectra were recorded on 660-IR (Varian, ATR mode) spectrometer. The signal of the molecular ion $[M-H]^-$ is reported in *m/z* units. UV/Vis absorption spectra were acquired on a Varian Cary 5000 UV/Vis-NIR (Varian) spectrophotometer with quartz cuvettes of 10 mm path length (QS Quartz Suprasil cells; Hellma Analytics). All compounds were measured in tetrahydrofuran in a wavelength region of 230–800 nm and under ambient conditions. The absorption maxima (λ_{max}) are reported in nm with the extinction coefficient (ε) in M^{-1} cm^{-1}.

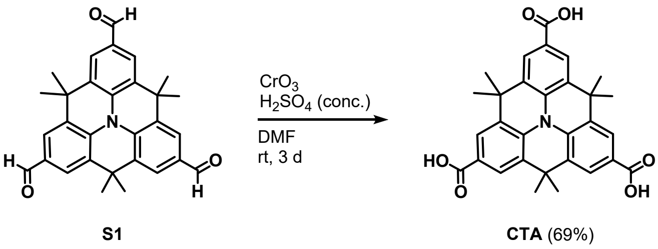

Scheme S1 Synthetic pathway towards tricarboxylic acid CTA. DMF = *N*,*N*-dimethylformamide.

Experimental procedure

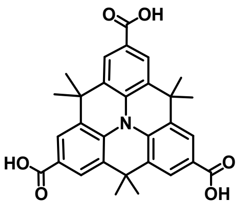

CTA

4,4,8,8,12,12-Hexamethyl-4*H*,8*H*,12*H*-benzo[1,9]quinolizino[3,4,5,6,7-*defg*]acridine-2,6,10-tricarbox-ylic acid (CTA)

To a solution of trialdehyde S1 (20.0 mg, 44.0 μmol) in DMF (2.00 mL), CrO_3 (26.0 mg, 260 μmol) and three drops of conc. H_2SO_4 were added under ambient conditions. The resulting dark green mixture was stirred at rt for 3 days. The reaction mixture was diluted with CH_2Cl_2 (5.00 mL), washed with sat. aq. $Na_2S_2O_5$ (2 × 10 mL), and extracted with CH_2Cl_2/EtOAc 2:1 (v/v, 3 × 20 mL). The combined organic phases were dried over $MgSO_4$, filtered, and the solvent was removed under reduced pressure. The crude product was dissolved in CH_2Cl_2/EtOAc 2:1 (v/v) and treated with hexanes to give CTA (15.0 mg, 30.1 μmol, 69%) as a pale yellow solid.

^{1}H NMR (300 MHz, $(CD_3)_2CO$): δ 11.20 (s, 3H), 8.17 (s, 6H), 1.73 (s, 18H) ppm.

^{13}C NMR (100 MHz, CD_3OD): δ 169.7, 136.0, 131.8, 127.6, 126.8, 36.8, 33.4 ppm.

UV/Vis (CH_2Cl_2): λ_{max} (ε in L mol^{-1} cm^{-1}) 252 (17300), 258 (17200), 313 (13600), 362 (22500) nm.

ESI-TOF HRMS (MeOH-MeCN-toluene): *m/z* calcd. for $C_{30}H_{26}NO_6$ [M–H]$^-$: 496.1766, found: 496.1766.

Nuclear Magnetic Resonance Spectra

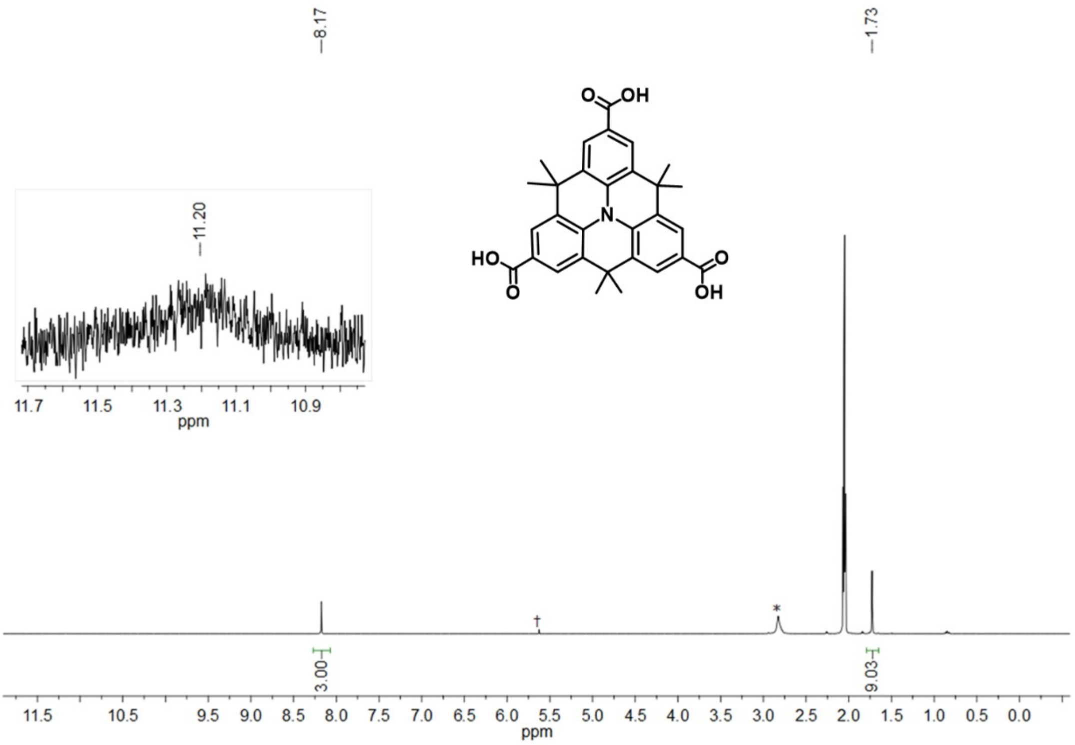

Figure S3 ^{1}H NMR spectrum of CTA (300 MHz, $(CD_3)_2CO$, rt);*water, †CH_2Cl_2.

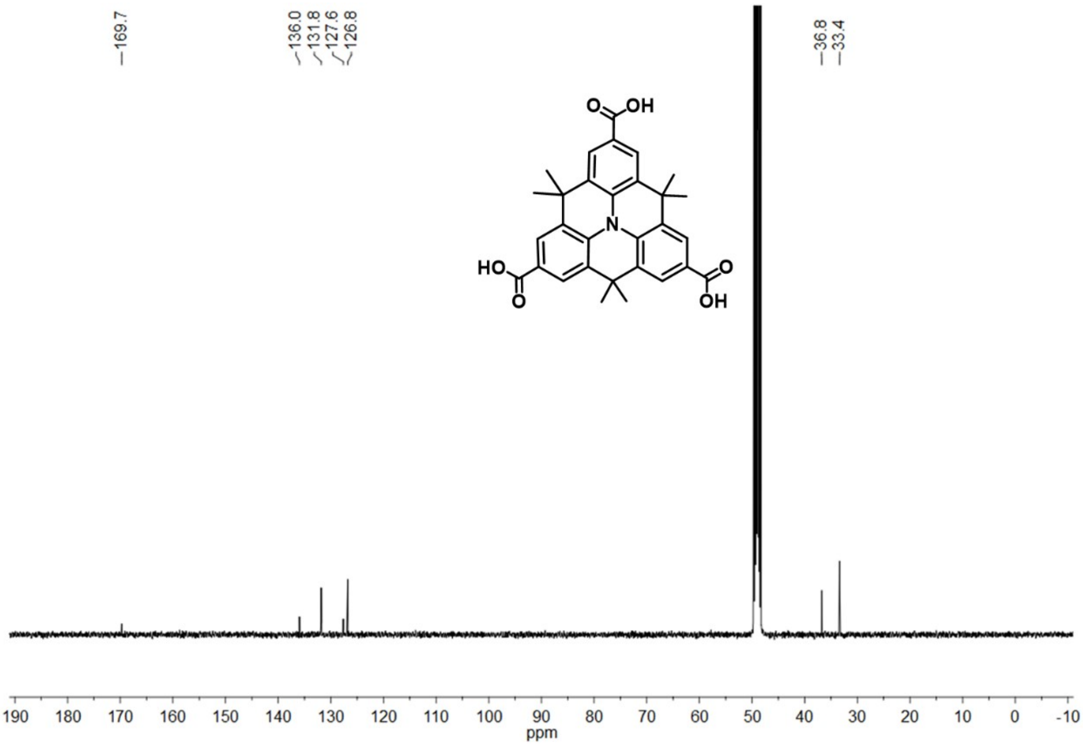

Figure S4 ^{13}C NMR spectrum of CTA (101 MHz, CD_3OD, rt).

STM experimental details

CTA flower structure:

The CTA flower structure was observed at the nonanoic acid – highly oriented pyrolytic graphite (NA-HOPG) interface when using the diluted solution only for a short amount of time of approximately three months. After that period, the CTA flower structure was not observed anymore. Attempts to observe the flower structure by either using the existing diluted solution or preparing fresh solutions were unsuccessful. Trying to reproduce the flower structure was done over a time period of 2.5 years.

Humidity effects on the switching behaviour:

The effect of humidity was identified based on differences observed in experiments conducted at different times of the year. The laboratory humidity was approximately 55 – 60% in summer and 20 - 25% in winter. Initially, it was observed that the speed of switching was different for the experiments conducted in summer vs. in winter. For subsequent experiments, we then carefully reviewed the experimental conditions and could confirm that the relative humidity indeed influences the switching behaviour. Between very low humidity (around 20%) and relatively high humidity (around 60%) a clear difference in the switching behaviour was found. However, when the differences in humidity are (around 10% difference), the changes in switching behaviour are subtle and less apparent. Although systematic experiments across the full 20–60% humidity range were not performed, the following general trend can be inferred: as humidity decreases, the transformation from CTA porous to close-packed phases takes longer.

References

[1] B. D. Gliemann, V. Strauss, J. Hitzenberger, P. O. Dral, F. Hampel. J.-P. Gisselbrecht, T. Drewello, W. Thiel, D. M. Guldi and M. Kivala, *Chem. Eur. J.*2017, **23**, 12353–12362.

[2] G. R. Fulmer, A. J. M. Miller, N. H. Sherden, H. E. Gottlieb, A. Nudelman, B. M. Stoltz, J. E. Bercaw and K. I. Goldberg, *Organometallics* 2010, **29**, 2176–2179.
